# Biogeographical Regionalization of Wine Yeast Communities in Greece and Environmental Drivers of Species Distribution at a Local Scale

**DOI:** 10.3389/fmicb.2021.705001

**Published:** 2021-06-30

**Authors:** Ioanna Chalvantzi, Georgios Banilas, Chrysoula Tassou, Aspasia Nisiotou

**Affiliations:** ^1^Hellenic Agricultural Organization “Dimitra”, Institute of Technology of Agricultural Products, Lykovrysi, Greece; ^2^Department of Wine, Vine and Beverage Sciences, University of West Attica, Athens, Greece

**Keywords:** yeasts, microbial diversity, microbial biogeography, microbial *terroir*, wine

## Abstract

Recent research has expanded our understanding on vineyard-associated fungal community assembly, suggesting non-random distribution and implicating regional differences in the wine *terroir* effect. Here, we focused on the culturable fraction of the fungal community that resides on grapes and determine wine quality, the so-called wine yeast populations. We aimed to analyze local-scale yeast community assemblages and to test whether the hypothesis of biogeographical patterns also applies to wine yeasts in particular. Surveying 34 vineyards across four main viticultural zones in Greece showed significant trends in vineyard-specific patterns. At a local scale, viticultural regions were also linked to distinct yeast community compositions. Importantly, major yeast populations directly related to wine fermentation contributed significantly to the delimitation of regions, highlighting their potential influence on the regionality of wine characteristics. In terms of the microbial *terroir* influence, yeast communities within an area were temporarily stable, which is critical for the regional character of the wine. Community structure could be explained only partially by environmental features. Maximum temperature, elevation, and net precipitation were the highest correlated variables with the yeast community biogeographic patterns. Finally, we also showed that certain environmental factors may drive the population size of specific yeast populations. The present results indicate that the wine yeast community has a geographical character at local scale, which is an important feature of the microbial *terroir* concept and thus for the wine industry.

## Introduction

The grape-associated yeast community is a significant component of the vine-wine system and is vastly associated with the quality of wine. Yeast community builds up concomitantly with grape berry development reaching densities of 3–5 Log CFU/g in ripe healthy grapes that may increase up to 7 Log CFU/g on damaged or botrytised grapes (Nisiotou and Nychas, 2007). Yeast species richness and relative species abundance are also in line with the stage of grape maturation. Grapes soon after the onset of ripening (veraison) are mainly colonized by basidiomycetous yeasts, such as *Cryptococcus* spp., *Rhodotorula* spp., *Sporobolomyces* spp., and the yeast-like fungus *Aureobasidium pullulans*, while thereafter they are gradually occupied by oxidative or weakly fermentative ascomycetous species, such as *Hanseniaspora* spp., *Metschnikowia* spp., *Pichia* spp., and *Candida* spp. (Barata et al., 2012). According to multiple reports, *Hanseniaspora uvarum* and *Metschnikowia pulcherrima* are the dominant species on mature healthy grapes, especially in warm climate regions (Nisiotou and Nychas, 2007; Settanni et al., 2012; Drumonde-Neves et al., 2017). To a smaller extent, it is possible to detect *Candida*, *Cryptococcus*, *Lachancea*, *Rhodotorula*, *Pichia*, and *Torulaspora* species, while *Saccharomyces cerevisiae*, the main wine fermenting yeast, is very scarce (Barata et al., 2012; Drumonde-Neves et al., 2017). Overripe, damaged, or botrytised grape berries support the growth of yeasts with high fermentative power and also of spoilages species like *Pichia* spp., *Zygoascus hellenicus*, *Zygosaccharomyces* spp., and *Torulaspora* spp. (Nisiotou et al., 2007; Nisiotou and Nychas, 2007; Barata et al., 2012).

The composition of grape microbial communities is affected by a number of factors including the sanitary state of grape berries, the climatic conditions, and the farming system (Nisiotou et al., 2007; Chavan et al., 2009; Li et al., 2010; Cordero-Bueso et al., 2011; Drumonde-Neves et al., 2017). Geographic separation has been suggested as another factor that may shape fungal grape microbial communities (Bokulich et al., 2014). Contrary to the Baas Becking hypothesis as “everything is everywhere but the environment selects,” it seems that the dispersal limitation may affect microbial communities. Recent data suggest a negative correlation between similarity of microbial communities and geographic distance, irrespective of the environmental factors, supporting thereby the existence of geospatial structures for microbial assemblages (Peay et al., 2010; Finkel et al., 2012; Cline and Zak, 2014; Morrison-Whittle and Goddard, 2015). Miura et al. (2017) showed that grape fungal community differences increased with geographic distance introducing thereby spatial processes as important factors in shaping yeast biogeographic patterns of vineyards.

Geographical patterns of grape-associated yeast communities form the basis for introducing a microbial notion to the *terroir* concept. *Terroir*, which expresses the influence of the place of origin in the special features of a food product, affects significantly consumer preferences and economic appreciation of wines (Van Leeuwen and Seguin, 2006). Still, the drivers and dimensions of wine “microbial *terroir*” have not been described in a comprehensive way. A growing body of research though indicates that wine yeast community patterns may correlate with the region of origin. For instance, geographical delineations of yeast communities have been documented in New Zealand vineyards (Gayevskiy and Goddard, 2012). Regional factors were also found to affect the fungal biodiversity within Californian sub-regions (Bokulich et al., 2014) or Portuguese appellations (Pinto et al., 2015). Similarly, Drumonde-Neves et al. (2017) observed significant differences among yeast communities in vineyards of five islands of the Azores Archipelago. Using next-generation sequencing (NGS) technology and metagenomics approaches, Taylor et al. (2014) and Morrison-Whittle and Goddard (2018) also detected significant differences in grape and must fungal communities at the genus level of different regions in New Zealand. Despite the advantages, due to the existence of numerous other fungi in the sample, NGS may provide proportionally more information on non-enological yeasts and fungi rather than to *S. cerevisiae* and non-*Saccharomyces* wine yeasts (Alexandre, 2020). However, these species contribute the most to wine fermentation and ultimately shape the wine phenotype and quality. Regional differences in yeast communities were further correlated to distinct wine metabolomes. Grape microbiota and wine metabolite profiles could distinguish viticultural area designations and individual vineyards within Napa and Sonoma Counties in California (Bokulich et al., 2016). Knight et al. (2015) showed that genetically differentiated regional populations of *S. cerevisiae* in New Zealand differentially affect wine phenotype. A positive correlation was also observed between phenotypic and genotypic diversity of regionally differentiated *Lachancea* thermotolerans vineyard strains in Greece (Banilas et al., 2016) as the first indication that the microbial *terroir* concept may also apply to strains of non-*Saccharomyces* wine yeast species.

Many questions about vineyard microbiota and their dispersion among vineyards and regions remain unanswered (Griggs et al., 2021). Here, we followed a culture-depended approach to isolate the grape-associated wine yeasts and to compare the yeast species assemblages within and between four major wine-producing regions in Greece (Peza in Crete, Santorini island, and the ancient viticultural zones of Nemea and Mantineia) over two consecutive years. The above regions form unique viticultural ecosystems as defined by inherent climatic and topographical features and historical viticultural practices. In the present study, the structure of wine yeast communities as a part of these peculiar ecosystems was questioned and potential drivers of regional yeast community assembly were investigated as per their role in the configuration of vine-wine microbial systems at local scale.

## Materials and Methods

### Grape Sampling

**Figure 1:**
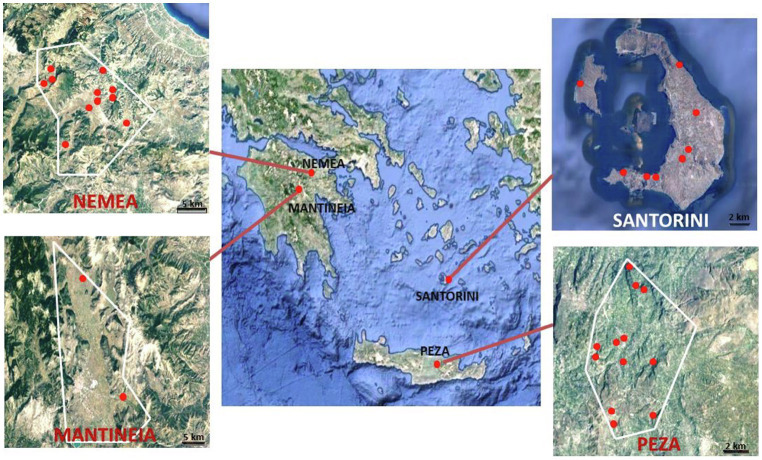
Maps of the vineyards sampled in four major PDO regions in Greece (Nemea and Mantineia in Peloponnese, Santorini island, and Peza in Crete). Images taken from Google Maps © 2021 TerraMetrics, Map data © 2021 Google.

Grape samples were collected from the following Protected Designation of Origin viticultural regions across southern Greece on an N-S maximum distance of ~ 370 km: Nemea in Peloponnese peninsula (11 vineyards, intermediate temperature zone), Mantineia plateau in Peloponnese peninsula (two vineyards, cool temperature zone), Santorini island (six vineyards in vintages A and B and three vineyards in vintage C; warm zone), and Peza in Crete island (10 vineyards in vintage A and four vineyards in vintage B; warm zone; Figure 1). Regions were sampled based on their size and their relative spatial heterogeneity. According to the National Inter-professional Organization of Vine and Wine portal for Greece’s vineyards and wines,^1^ Nemea and Peza are the largest viticultural regions (30 km^2^ cultivated vineyards each) and present the most heterogeneous landscapes among the areas surveyed. Nemea is divided into three altitude zones, the lowland (at elevation of 260–350 m), the semi-mountainous (350–600 m), and the mountainous (600–800 m). In Peza, the vines are spread amphitheatrically on low hills with varying slope up to an altitude of 700 m (mountainous and semi-mountainous vineyards). Santorini has 15 km^2^ of vineyards, located in the central and the southern parts of the island. Mantineia is the smallest viticultural region (10 km^2^), which presents a uniform landscape, located on a mountainous plateau at an altitude of 650 m. Two to nine sampling plots per vineyard were surveyed as previously described (Chalvantzi et al., 2020) giving a total of 156 grape samples. Grapes were placed into sterile plastic bags, transferred to the laboratory and crushed with a stomacher.

### Analytical Determinations

Reducing sugars, total acidity, and pH determinations were estimated by the methods described in the “Compendium of International Methods of Analysis of Musts and Wines,” International Organization of Vine and Wine (OIV, 2015).

### Isolation and Identification of Yeasts

For the enumeration and isolation of yeasts, grape juice serial dilutions were plated into Wallerstein laboratory nutrient agar, lysine medium agar, and ethanol sulfite agar supplemented with 100 mg/l biphenyl and 100 mg/l chloramphenicol. Colonies were randomly selected from plates, purified by streak plating technique and examined microscopically. Isolates were stored at −80°C until further analysis. Cells were collected from plates with a sterile yellow tip and suspended in 3 μl of NaOH 0.02 M solution. Cell suspensions were then incubated at 99°C for 10 min. The ITS-5.8S rDNA region was PCR-amplified using the primers ITS1 and ITS4 (White et al., 1990). Sizes of amplicons were determined using a 100-bp molecular weight marker in 1.2% (w/v) agarose gels. For species-level assignment 500 ng of PCR products were digested by HinfI, HaeIII, or HhaI according to Esteve-Zarzoso et al. (1999) and analyzed on 3% (w/v) agarose gel. Further digestions by the restriction enzymes DdeI and DraI were performed to differentiate between *Hanseniaspora* and *Candida* genera as previously suggested (Nisiotou et al., 2007). PCR products of representative yeast isolates per distinct restriction pattern were purified using the QIAquick PCR purification kit and directly sequenced (Macrogen).^2^ BLAST searches of sequences were performed at the NCBI/GenBank database, and the Clustal X software was used to construct multiple sequence alignments.

### Climate Data

Climatic data were collected from weather stations located in the vineyard regions. Daily measurements for average high temperature, average low temperature, average temperature, maximum temperature, minimum temperature, net precipitation, average wind speed, maximum wind speed, and average maximum wind speed were obtained.

### Statistical Analysis

Differences in yeast community composition between regions, vineyards, or vintages were tested with Analysis of Similarity (ANOSIM) on square-root transformed species incidences using the Bray–Curtis distance matrices. ANOSIM R statistic close to the maximum limit of one implies robust group separation rejecting the null hypothesis *R* = 0 (no differences between groups), while negative values of *R* show higher intra-group than inter-group diversity. Values of *p* < 0.05 indicate significantly different level between groups. When the null hypothesis was rejected, pairwise values of *R* were examined to reveal major differences between regions. Permutational multivariate analysis of variance (PERMANOVA) tests with 999 permutations was also applied to test significant differences between sample groups using Bray–Curtis similarity measure, where higher values of *F* statistic indicate greater between-groups variations. Cluster analysis dendrograms were constructed based on Bray–Curtis similarity matrices to test clustering of yeast communities according to the vineyard or the region of origin. Principal coordinate analysis (PCoA) based on Bray–Curtis distances was applied, as an unconstrained method, to depict resemblances of samples in a two-dimensional space. Following PCoA, canonical discriminant analysis (CDA), a constrained ordination method where the groups are defined *a priori*, was used to produce an ordination plot and compare samples of different regions against species abundances, according to Anderson and Willis (2003). Ordination by non-metric multidimensional scaling (nMDS) was also used to reflect yeast community resemblances by projecting the pairwise dissimilarity between objects in a two-dimensional space as accurately as possible (Legendre and Legendre, 1998; Ramette, 2007). Mantel test (Spearman’s rank correlations with 999 permutations), a widely used test to compare matrices calculated for the same objects but from independent data sets (Ramette, 2007), was applied to determine the correlation between geographic distance of regions and yeast community (dis)similarity matrices. Significant differences between environmental conditions of different regions or vintages were tested with ANOVA. BEST was applied as described (Clarke and Gorley, 2015) to identify the most important environmental factors that drive community differences across regions. Distance-based linear model (DistLM) marginal test was applied to evaluate correlation of the environmental factors with the yeast community structure patterns and to test significance. Distance-based redundancy analysis (db-RDA) was used for the ordination and visualization of DistLM (Anderson et al., 2008). Partial least squares regression (PLSR) model was applied to demonstrate associations between environmental variables (predictor variables X) and specific yeast populations (response variables Y) and to detect particular climatic factors that drive the distribution of yeast populations across regions. All statistical tests were performed using the software PRIMER Version 7 with PERMANOVA+^3^ except for PLSR and CDA that were conducted with JMP.^4^

## Results

### Wine Yeast Species Abundance and Community Heterogeneity

A total of 156 grape samples derived from 34 vineyards covering highly important viticultural regions across Greece were analyzed (Figure 1). Yeast counts differed significantly among regions (*p* < 0.001). Samples from the Santorini island exhibited the highest yeast counts (5.85 ± 1.06 Log CFU/ml) followed by Mantineia (5.49 ± 0.30 Log CFU/ml), Peza (4.54 ± 0.98 Log CFU/ml), and Nemea (3.66 ± 1.10 Log CFU/ml). Species richness was also significantly higher (*p* < 0.05) in Santorini (16 species) compared to the other regions.

**Figure 2:**
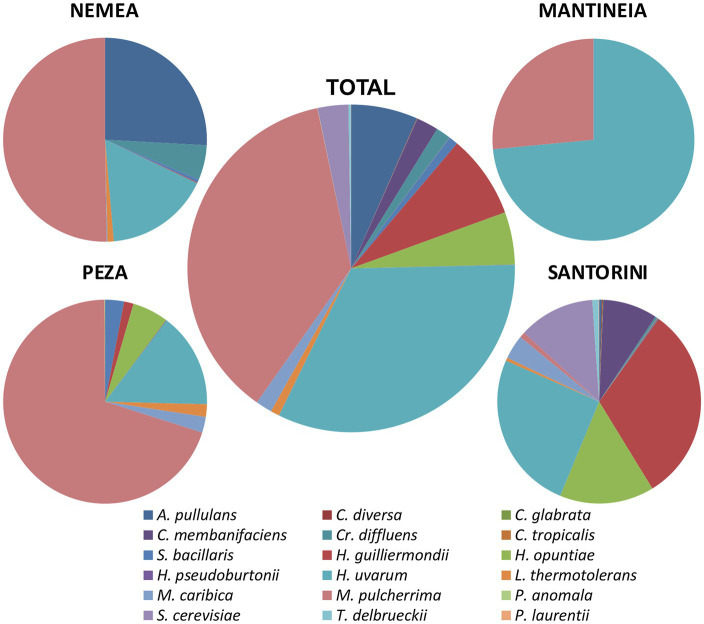
Species diversity and relative abundance of yeasts in different winegrowing regions.

Yeast isolates were analyzed by PCR-RFLP analysis of the 5.8S-ITS rDNA region. Restriction enzyme banding profile comparisons between isolates and published strains combined with sequence analysis of the 5.8S-ITS region assigned groups of isolates to 18 different yeast species, namely, *Aureobasidium pullulans*, *Candida diversa*, *C. glabrata*, *C. membranifaciens*, *C. tropicalis*, *Cryptococcus diffluens*, *H. uvarum*, *H. guilliermondii*, *H. opuntiae*, *Hyphopichia pseudoburtonii*, *Lachancea thermotolerans*, *Meyerozyma caribbica*, *M. pulcherrima*, *Papiliotrema laurentii*, *Pichia anomala*, *S. cerevisiae*, *Starmerella bacillaris*, and *Torulaspora delbrueckii*. Except for *P. laurentii*, the majority of the species belonged to Ascomycota, including species that significantly contribute to the alcoholic fermentation of grape must. Figure 2 shows the species diversity and their relative abundance in the different viticultural regions studied. *M. pulcherrima* was the most frequently isolated yeast (Figure 2; 37%). *M. pulcherrima* was highly encountered in Peza (70%), followed by Nemea (50%). It was moderately abundant in Mantineia (27%), but scarcely detected in Santorini (1%). *H. uvarum* was the second most encountered species across vineyards. It was highly abundant in Mantineia (74%) and moderately found in other regions, i.e., 25% in Santorini, 17% in Nemea, and 15% in Peza.

Only five out of a total 18 species were inter-regionally encountered, i.e., *S. bacillaris*, *H. guilliermondii*, *H. uvarum*, *M. caribbica*, and *M. pulcherrima*. Several species were associated exclusively with Santorini, namely, *C. diversa*, *C. glabrata*, *C. membranifaciens*, *P. laurentii*, *C. tropicalis*, *S. cerevisiae*, and *T. delbrueckii*, while *H. pseudoburtonii* and *P. anomala* were solely encountered in Peza.

### Inter-Regional Yeast Community Similarities

We further asked whether there is any difference between the yeast communities of different viticultural regions. Both ANOSIM and PERMANOVA tests indicated that species composition of yeast communities differed significantly among regions (ANOSIM: *R* = 0.401, *p* = 0.001; Table 1). ANOSIM pairwise tests revealed that all pairs of regions shared significantly dissimilar communities, except for the nearby Mantineia and Nemea regions, both located in Peloponnese, and Mantineia *vs*. Santorini (Table 2).

**Table 1:** Analysis of Similarity (ANOSIM) and Permutational multivariate analysis of variance (PERMANOVA) of the factors “region,” “vintage,” and “vineyard” on wine yeast diversity patterns.

Region	Vintage	Factor	ANOSIM		PERMANOVA	
			*R*	*p*	*F*	*p*
All	All	Region	0.401	0.001^*^	20.339	0.001^*^
All	A	Region	0.525	0.001^*^	14.678	0.001^*^
All	B	Region	0.480	0.001^*^	12.207	0.001^*^
All	All	Vintage	0.043	0.138	3.4479	0.014^*^
Nemea	All	Vintage	−0.038	0.834	0.1001	0.908
Peza	All	Vintage	−0.022	0.506	0.6741	0.517
Santorini	All	Vintage	0.379	0.001^*^	6.3601	0.001^*^
Mantineia	All	Vintage	0.000	0.400	0.0758	1.000
All	All	Vineyard	0.508	0.001^*^	12.525	0.001^*^
Nemea all	All	Vineyard	0.388	0.001^*^	6.2636	0.001^*^
Nemea	A	Vineyard	0.605	0.030^*^	8.5558	0.005^*^
Nemea	B	Vineyard	0.260	0.104	3.3663	0.042^*^
Santorini	A, B	Vineyard	0.471	0.001^*^	12.350	0.001^*^
Santorini	A	Vineyard	0.820	0.001^*^	13.015	0.001^*^
Santorini	B	Vineyard	0.456	0.044^*^	2.3175	0.055
Peza	A	Vineyard	0.121	0.110	3.1363	0.035^*^

* *p* < 0.05.

**Table 2:** ANOSIM pairwise tests for differences on yeast community composition between regions.

	Santorini	Nemea	Peza	Mantineia
**Santorini**		**0.44**	**0.695**	**0.106**
**Nemea**	*0.001*		**0.149**	**0.141**
**Peza**	*0.001*	*0.002*		**0.627**
**Mantineia**	*0.11*	*0.1*	*0.001*	

Values of *R* are indicated in bold and values of *p* in italic. Statistically significant differences are indicated with beige color.

**Figure 3:**
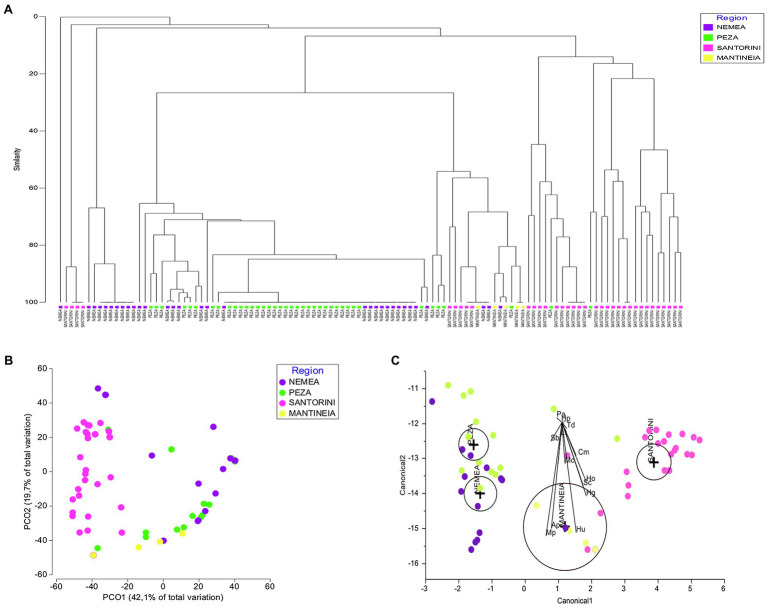
Vineyard-associated yeast communities of Mantineia, Nemea, Peza, and Santorini viticultural regions. **(A)** Plot Bray–Curtis similarity dendrogram comparing yeast communities of the four regions (Cophenetic correlation: 0.93), **(B)** Principal coordinate analysis (PCoA) based on Bray–Curtis distances depicting resemblance of samples in a two-dimensional space, and **(C)** Canonical discriminant analysis plot comparing grape samples of different regions against yeast species abundances. The diameter of the circle represents a 95% of confidence interval for the mean. Significantly different groups have non-intersecting circles. Ap, *Aureobasidium pullulans*; Cd, *Candida* diversa; Cg, *C. glabrata*; Cm, *C. membranifaciens*; Ct, *C. tropicalis*; Cd, *Cryptococcus diffluens*; Hu, *Hanseniaspora uvarum*; Hg, *H. guilliermondii*; Ho, *H. opuntiae*; Hp, *Hyphopichia* *pseudoburtonii*; Lt, *Lachancea thermotolerans*; Mc, *Meyerozyma caribbica*; Mp, *Metschnikowia pulcherrima*; Pl, *Papiliotrema laurentii*; Pa, *Pichia anomala*; Sc, *Saccharomyces cerevisiae*; Sb, *Starmerella bacillaris*; and Td, *Torulaspora delbrueckii*.

A Bray–Curtis similarity dendrogram was constructed (Cophenetic correlation: 0.93) to compare yeast communities of the four regions (Figure 3A). Most samples of the same origin were placed together and rather separated from those of other regions, supporting the results of both ANOSIM and PERMANOVA. Nevertheless, a close relationship was observed for some samples from Nemea and Peza. PCoA based on Bray–Curtis dissimilarities of species composition among the samples depicted clustering of samples from the same region, with Nemean samples being more dispersed than others and also related to samples from Peza (Figure 3B). CDA highlighted regional yeast species associations (Figure 3C). Regions had non-intersecting circles suggesting that groups were significantly different. The species that strongly discriminate between regions include *M. pulcherrima* and *S. bacillaris* (strongly associated with Nemea and Peza), *A. pullulans* (Nemea), *H. uvarum* (Mantineia), and *H. opuntiae* and *H. guilliermondii* (Santorini).

### Intra-Regional Yeast Community Similarities

**Figure 4:**
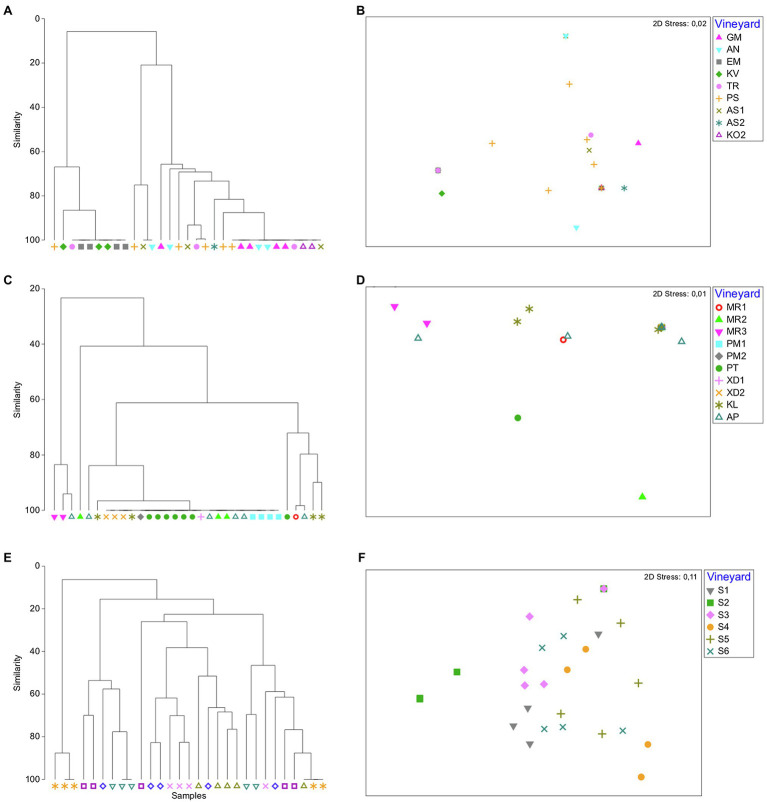
Bray–Curtis non-metric multidimensional scaling plots and dendrograms of yeast community vineyard samples in Nemea **(A,B)**, Peza **(C,D)**, and Santorini **(E,F)**, respectively.

We also compared the yeast communities within regions, where multiple vineyards were sampled across vintages, i.e., Nemea and Santorini regions (vintages A and B) and Peza region (vintage A). Overall, significant trends of vineyard-specific patterns were revealed by both ANOSIM and PERMANOVA tests (ANOSIM: *R* = 0.508, *p* = 0.001; PERMANOVA: *R* = 12.525, *p* = 0.001; Table 1). Significant dissimilarities were recorded between vineyards in Santorini island, across both vintages (*R* = 0.471, *p* = 0.001; Table 1 and Figure 4). Significant differences were also detected between vineyards in Nemea, although dissimilarities were lower in vintage B than A (ANOSIM: *R* = 0.388, *p* = 0.001; Table 1 and Figure 4). Although there was not a clear differentiation between Peza vineyards, distinct community patterns were detected between the northern and the southern vineyards. The southern vineyards were characterized by the complete dominance of *M. pulcherrima*, while the northern vineyards showed much higher biodiversity. By applying nMDS and hierarchical clustering dendrograms grouping of samples according to their vineyard of origin was detected in Peza and Santorini, but it was less obvious in Nemea. We further asked whether there was any structure according to the geographical distance of vineyards but Mantel test did not support such a correlation (*p* ≥ 0.34).

### Similarities of Yeasts Communities Over Time

**Figure 5:**
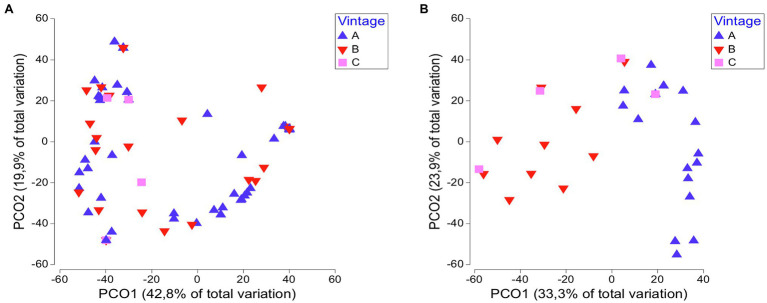
PCoA score plot of yeasts communities across vintages: **(A)** all regions and **(B)** Santorini.

We examined whether the yeast communities in different viticultural regions are temporarily stable. By applying ANOSIM and PERMANOVA tests, no significant differences were observed over vintages (ANOSIM: *R* = 0.043, *p* = 0.138; Table 1). The key yeast species in regional yeast communities were steadily established across vintages in each region, except for Santorini. There was a pronounced differentiation among vintages as per the dominant species in Santorini island, with *H. opuntiae*, *H. guilliermondii*, and *H. uvarum* prevailing in vintages A, B, and C, respectively. Similar results were obtained with PCoA. Overall, there was not a clear separation of samples from different vintages, suggesting that the yeast communities remained rather unchanged over time (Figure 5A). Only in Santorini, the samples from different vintages were placed separately, particularly those of vintage A (Figure 5B).

### Effect of Environmental Conditions

We further asked whether the environmental conditions (climatic and grape must characteristics) affect regional yeast community delimitation. Significant differences were detected between regions in the majority of the environmental conditions with the exception of the average high and maximum temperatures and the sugar content of grape juice (ANOVA; Supplementary Table S1). Conversely, the environmental conditions did not show statistically significant yearly fluctuations within regions, with a few exceptions (Supplementary Table S2). Environmental factors were moderately related with yeast diversity similarity at Rho = 0.386 (*p* = 0.0001, Spearman rank correlation, 999 permutations).

A BEST was applied to identify the most important environmental factors that drive community differences across regions. BEST showed that among the different environmental factors the maximum temperature could better explain yeast community differentiation (Supplementary Table S3). The maximum temperature along with the elevation was the most highly correlated couple of variables with the yeast community biogeographic patterns (Supplementary Table S3).

**Figure 6:**
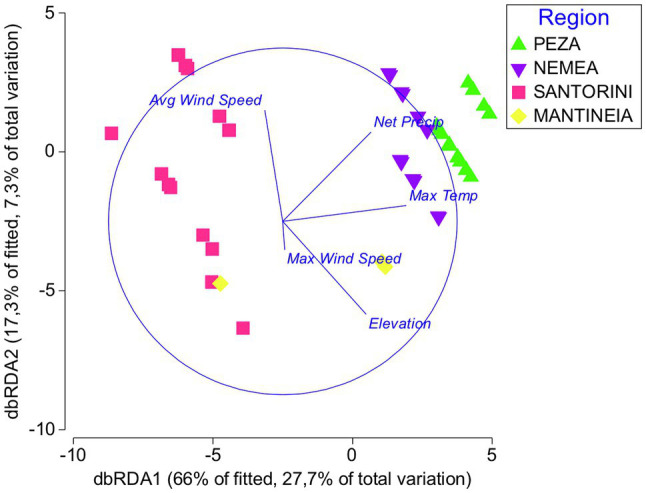
Distance-based redundancy analysis showing the relationships between yeast communities in different regions (Peza, Nemea, Santorini, and Mantineia) and most important environmental factors (Average Wind Speed, Net Precipitation, Elevation, Maximum Temperature, and Max Wind Speed).

According to the distance-based linear model marginal test, there was a significant correlation between yeast assemblages and each of the environmental variables analyzed, except for the sugar content (Supplementary Table S4). Maximum temperature, net precipitation, and elevation were the variables that showed the highest values of *F*, each explaining 19–23% of the total yeast diversity. Stepwise sequential tests also highlighted the importance of average wind speed and maximum wind speed, which along with average maximum wind speed and average high temperature cumulatively explained approximately 50% of the variation (Supplementary Table S4). db-RDA further justified the variables maximum temperature, net precipitation, elevation, average wind speed, and maximum wind speed as the most critical environmental factors that shape yeast community structures in different viticultural regions. The combination of these factors explained 83.3% of the total variation (Figure 6).

**Figure 7:**
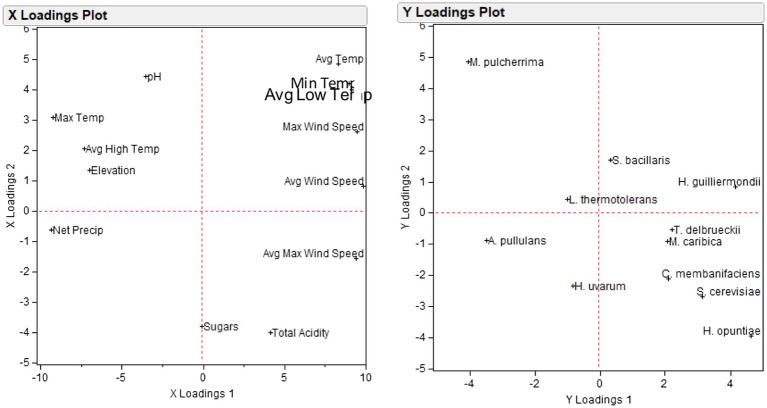
Loading plots of partial least squares regression analysis PLSR of 13 environmental variables (X loadings plot) and 11 yeast populations (Y loadings plot).

A partial least squares regression model (PLSR) was applied to demonstrate the association between the set of environmental variables, and the yeast species in order to identify which climatic factors may drive specific yeast populations across regions and vintages. Among the different yeast populations, 11 species explained more than 10% of the variation in the first two components and were thus retained for the construction of the PLSR model. Projections revealed highly covariable relationships between specific environmental factors and yeast species (Figure 7). In particular, *M. pulcherrima* was strongly associated with pH and maximum temperature, *H. guilliermondii* with average wind speed, *A. pullulans* with net precipitation, *H. uvarum* with net precipitation and sugars, and *H. opuntiae* with total acidity.

## Discussion

Wine is an important agricultural commodity that exhibits differential regional phenotypes, attributed to the *terroir* effect. The geographical variation of wines based on their chemical and sensory profiles has been well documented (Fischer et al., 1999; Pereira et al., 2005; Son et al., 2009; López-Rituerto et al., 2012; Robinson et al., 2012). Although differential wine phenotypes among distinct regions establish the *terroir* hypothesis, the connective causes of the wine *terroir* concept are still unclear (Van Leeuwen and Seguin, 2006). In this context, the metabolic impact of indigenous yeasts on wine organoleptic features has been documented (Swiegers et al., 2005; Ciani et al., 2010; Nisiotou et al., 2018; Sgouros et al., 2018; Nisiotou et al., 2019). However, whether wine yeast communities are geographically structured causing thereby regional distinctiveness of wines remains to be elucidated.

Here, we surveyed 34 vineyards of four major viticultural zones in Greece. Viticultural zones showed a relatively high inter-regional heterogeneity, compared to analogous studies (Gayevskiy and Goddard, 2012; Drumonde-Neves et al., 2017). This variation can be attributed to the extremely diverse landscape structure, which includes plateau, islands, and hilly regions that, despite their geographic proximity, belong to distinct climatic zones. *M. pulcherrima* dominated the vineyards of Peza. In Nemea vineyards, *M. pulcherrima* and *A. pullulans* were the most common yeasts, while *H. uvarum* dominated Mantineia plateau. Either *H. guilliermondii* or *H. opuntiae* (depending on the vintage) was prevailing Santorini island. Differences were also detected in minor populations and only five out of 18 species were inter-regionally encountered. Notably, several species were region-specific. It is worth mentioning that the same amounts of *H. uvarum* domination (70%) were previously found in Mantineia plateau (Nisiotou et al., 2007).

Species composition and abundance differed from other wine-producing regions. For instance, a different and more unvarying yeast community composed largely by *Rhodotorula glutinis* and *A. pullulans* was observed in three wine-producing regions of New Zealand’s vineyards covering about the same area as in the present study (Gayevskiy and Goddard, 2012). *H. uvarum*, *M. pulcherrima*, and *Pichia terricola* were the dominant species in wine-producing regions of the Azores Archipelago of the North Atlantic Ocean (Drumonde-Neves et al., 2017). Several other studies conducted across the world have identified diverse yeast community compositions and abundances. For instance, *H. uvarum*, *M. pulcherrima*, *L. thermotolerans*, and *Rhodotorula* sp. were the most frequently isolated yeasts in four South African vineyards (Jolly et al., 2003). *H. uvarum*, *M. pulcherrima*, and *A. pullulans* were the most abundant populations in grapes from 10 vineyards in western Sicily (Settanni et al., 2012). More research aimed at firmly linking a viticultural area with the local yeast community will allow identifying global variations and better spot worldwide differences.

At the local scale, each vineyard was found to have a comparatively homogeneous yeast community, which was distinguished from nearby vineyards in the same viticultural region. Differences in the structure of fungal communities between vineyards have been suggested previously (Miura et al., 2017), although the relative information is quite scarce. Grape yeast biota has been also proposed to rely on spatial fluctuation even at the vineyard scale (Setati et al., 2012; Liu et al., 2019). The intra-vineyard fungal diversity has been ascribed to microclimate variability, but there is no experimental validation for this hypothesis. Usually, a vineyard forms a relatively uniform environmental niche as it is subjected to the same pedoclimatic conditions and farming treatments. It is plausible to conclude therefore that, at least for yeast populations, single vineyards may support the growth of a relatively similar species composition (Sabate et al., 2002; Vigentini et al., 2015). We are not certain whether vineyard-associated microbial patterns present a distance-dependent model at a local scale. Here, however, it was shown that the geographical distance of vineyards within a region did not correlate with the level of yeast community dissimilarity.

Previous metagenomics studies suggested a non-random distribution of vineyard-associated fungal or bacterial populations, with regional and environmental features being among the most critical factors affecting community assembly (Bokulich et al., 2014; Morrison-Whittle and Goddard, 2018). However, it is not clear whether the above applies to wine yeast populations alone, which constitute a subset of the total fungal community. As a first goal of this study, we conducted multiple grape berry sampling to acquire a comprehensive picture of the spatio-temporal structure of wine yeast communities in the major viticultural zones of Greece. We targeted the culturable wine yeast community rather than fungal populations unrelated to wine production. To this end, it was clearly shown that yeast communities differ significantly at local scale. The differences were ascribed to both the dominant and the minor populations. Viticultural regions were associated with diverse community compositions, which might affect regional characteristics of wines, introducing thereby a microbial notion to wine *terroir*. Importantly, major players in wine fermentation, like, *H. uvarum*, *H. opuntiae*, *H. guilliermondii*, and *M. pulcherrima* significantly contributed to delimitation among viticultural areas. These yeasts have been shown to confer significant metabolic impact on wine quality (Ciani et al., 2010; Nisiotou et al., 2018, 2019; Sgouros et al., 2018).

To assign a microbial aspect to wine *terroir*, a temporal stability for regional yeast communities is essential (Chalvantzi et al., 2020). However, the extent of year-to-year fluctuation in the wine yeast community assembly of a viticultural region has hardly been studied. It was previously shown that vintage may significantly affect the vineyard-associated yeast biodiversity (Sabate et al., 2002; Bokulich et al., 2014; Vigentini et al., 2015). As opposed, we did not detect any significant difference between vintages in the different regions surveyed, except for the island of Santorini. This further justifies our conclusion about the distinctiveness of regional wine yeast communities, in that the differences detected are not stochastic but show temporal stability.

The inherent climatic and topographical features of viticultural regions form the basis of the wine *terroir*. It has been proposed that grapevine microbiomes have a spatial distribution that corresponds to environmental conditions both between and inside vineyards (Griggs et al., 2021). It is unclear, however, which environmental factors affect the grape-associated regional wine yeast communities and explain the magnitude of biodiversity. Here, it was shown that the environmental factors could explain a part of the total yeast biodiversity. BEST-tests revealed maximum temperature, elevation, and net precipitation as the highest correlated variables with the yeast community biogeographic patterns. Similarly, environmental features were previously shown to affect the grape-associated microbial communities, but poor R^2^ coefficients suggested weak feature importance (Bokulich et al., 2014). Here, distance-based linear model marginal test showed significant correlation between yeast assemblages and each of the environmental variables individually. Maximum temperature, net precipitation, and elevation showed the highest values of *F*. Factually, elevated altitude forms a hostile environment for microorganisms by exposing cells to ultraviolet (UV) radiation, desiccation, and low temperatures and it was recently shown to significantly affect airborne fungal communities (Tanaka et al., 2019). Net precipitation is another important environmental feature affecting microbial biodiversity as recently shown for soil fungi global niche differentiation (Bahram et al., 2018). We also showed that certain environmental factors may drive the population size of specific yeast populations, like pH and maximum temperature of *M. pulcherrima*, average wind speed of *H. guilliermondii*, and total acidity of *H. opuntiae*. Net precipitation was found to be highly correlated with *A. pullulans* and *H. uvarum*. Precipitation levels were previously shown to correlate with the abundance of *Metschnikowia*, *Torulaspora*, and *Saccharomyces* yeast species in grapes from different Chilean valleys (Jara et al., 2016). Given the importance of these species in wine fermentation, it is reasonable to assume that variations in certain climatic conditions can contribute to distinct wine phenotypes by altering specific wine yeast populations.

Our results here support the hypothesis that different regions harbor distinct microbial communities, which was shown to apply specifically to the wine-associated yeast populations. We also highlight the implication of climatic features in regional wine yeast biogeography. The fact, however, that major environmental features, like climatic and grape must characteristics, could explain only a part of biogeography points to the complexity of the factors driving wine yeast community composition. Other regional factors, including modes of yeast dispersion parameters, landscape composition or biotic interactions may also interfere in community assembly.

## Data Availability Statement

The original contributions presented in the study are included in the article/Supplementary Material, and further inquiries can be directed to the corresponding author.

## Author Contributions

AN designed the study, performed experiments, analyzed data, and wrote the paper. IC conducted experiments and analyzed data. CT contributed to data analysis and writing the paper. GB analyzed data and contributed to writing the paper. All authors contributed to the article and approved the submitted version.

### Conflict of Interest

The authors declare that the research was conducted in the absence of any commercial or financial relationships that could be construed as a potential conflict of interest.

## Footnotes

^1^https://winesofgreece.org

^2^http://www.macrogen.com

^3^https://www.primer-e.com

^4^https://www.jmp.com
