# Supplementary material for: Biogeographical Regionalization of Wine Yeast Communities in Greece and Environmental Drivers of Species Distribution at a Local Scale
Source: Front Microbiol. 2021 Jun 30;12:705001. doi: 10.3389/fmicb.2021.705001 (PMC8278314; doi:10.3389/fmicb.2021.705001)
Supplement: Supplementary file 1 [file Table_1.DOCX]

Supplementary Material

# Supplementary Tables

**Supplementary Table S1.** ANOVA results of environmental factors between regions

| **Factor** | **F** | ***P*** |
| --- | --- | --- |
| Average Temperature | 3.66 | =0.0148* |
| Average High Temperature | 0.22 | =0.8805 |
| Average Low Temperature | 22.81 | <0.0001* |
| Maximum Temperature | 0.92 | =0.4315 |
| Minimum Temperature | 23.67 | <0.0001* |
| Net Precipitation | 4.04 | =0.0092* |
| Average Wind Speed | 120.58 | <0.0001* |
| High Wind Speed | 21.57 | <0.0001* |
| Average High Wind Speed | 24.69 | <0.0001* |
| Altitude | 30.70 | <0.0001* |
| pH | 19.18 | <0.0001* |
| Sugars | 4.40 | =0.0054 |
| Total acidity | 8.95 | <0.0001* |

**Supplementary Table S2.** ANOVA results of environmental factors between vintages

| **Factor** | **Region** | **F** | ***p*** |
| --- | --- | --- | --- |
| Average Temperature | Nemea | 0.0191 | 0.8914 |
|  | Santorini | 0.1422 | 0.8680 |
|  | Mantineia | 0.0227 | 0.8816 |
|  | Peza | 0.0001 | 0.9915 |
| Average High Temperature | Nemea | 0.0168 | 0.8981 |
|  | Santorini | 0.1344 | 0.8747 |
|  | Mantineia | 0.0253 | 0.8747 |
|  | Peza | 0.0011 | 0.9734 |
| Average Low Temperature | Nemea | 0.0000 | 0.9961 |
|  | Santorini | 0.1921 | 0.8261 |
|  | Mantineia | 0.0350 | 0.8528 |
|  | Peza | 0.0005 | 0.8976 |
| Maximum Temperature | Nemea | 0.0161 | 0.9002 |
|  | Santorini | 0.0203 | 0.9799 |
|  | Mantineia | 0.0280 | 0.8681 |
|  | Peza | 0.0488 | 0.8273 |
| Minimum Temperature | Nemea | 0.1303 | 0.7216 |
|  | Santorini | 0.5117 | 0.6042 |
|  | Mantineia | 0.1222 | 0.7288 |
|  | Peza | 0.1474 | 0.7047 |
| Net Precipitation | Nemea | 0.0510 | 0.8233 |
|  | Santorini | 0.4684 | 0.6301 |
|  | Mantineia | 0.3555 | 0.5550 |
|  | Peza | 0.2863 | 0.5980 |
| Average Wind Speed | Nemea | 2.1238 | 0.1592 |
|  | Santorini | 1,1124 | 0.3408 |
|  | Mantineia | 4.3906 | 0.0437* |
|  | Peza | 11.1344 | 0.0031* |
| High Wind Speed | Nemea | 0.0 | 1.0000 |
|  | Santorini | 0,0239 | 0,9764 |
|  | Mantineia | 7,7723 | 0,0107* |
|  | Peza | 2.3435 | 0.1407 |
| Average High Wind Speed | Nemea | 2.1234 | 0.1592 |
|  | Santo | 0.5231 | 0.5975 |
|  | Mantineia | 2.2637 | 0.1467 |
|  | Peza | 4.0803 | 0.0563 |
| pH | Nemea | 0.6706 | 0.4164 |
|  | Santo | 13.3923 | <0.001* |
|  | Mantineia | 1.5351 | 0.2703 |
|  | Peza | 0.2147 | 0.6452 |
| Sugars | Nemea | 1.7400 | 0.1926 |
|  | Santo | 8.9856 | 0.0008 |
|  | Mantineia | 2.4093 | 0.1813 |
|  | Peza | 1.6912 | 0.1997 |
| Total Acidity | Nemea | 2.0520 | 0.1577 |
|  | Santo | 40.5870 | <0.001* |
|  | Mantineia | 1.1371 | 0.3350 |
|  | Peza | 0.0377 | 0.8468 |

**Supplementary Table S3.** Best variable rank correlation (BEST) results of environmental factors affecting community assembly.

| No. of variables | Correlation | Selections |
| --- | --- | --- |
| 1 | 0.529 | Max T |
| 2 | 0.592 | El, Max T |
| 3 | 0.577 | El, Max T, Ne |
| 4 | 0.570 | El, Avg H, Max T, Avg M |
| 5 | 0.562 | El, Avg H, Max T, Ne, Avg M |

El = Elevation, To = Total Acidity, Avg T = Average Temperature, Avg H = Average High Temperature, Avg L = Average Low Temperature, Max T = Max Temperaure, Mi = Minimum Temperature, Ne = Net Precipitation, Avg W = Average Wind Speed, Max W = Maximum Wind Speed, Avg M = Average Maximum Wind Speed

**Supplementary Table S4.** Distance-based linear model (DLM) marginal test based on the environmental variables used to evaluate correlation with the yeast community structure patterns.

| Variable | SS (trace) | Pseudo-F | *p* | Proportion of variation |
| --- | --- | --- | --- | --- |
| Elevation | 1330.7 | 24.526 | 0.001 | 0.1865 |
| pH | 487.52 | 7.8462 | 0.001 | 0.0683 |
| Sugars | 99.508 | 1.5132 | 0.180 | 0.0139 |
| Total Acidity | 357.59 | 5.6447 | 0.001 | 0.0501 |
| Avg Temp | 412.82 | 6.5702 | 0.001 | 0.0579 |
| Avg High Temp | 878.27 | 15.017 | 0.001 | 0.1231 |
| Avg Low Temp | 492.74 | 7.9365 | 0.001 | 0.0691 |
| Max Temp | 1642.3 | 31.986 | 0.001 | 0.2301 |
| MinTemp | 415.16 | 6.6097 | 0.002 | 0.0582 |
| Net Precip | 1348.3 | 24.926 | 0.001 | 0.1889 |
| Avg Wind Speed | 781.65 | 13.162 | 0.001 | 0.1095 |
| Max Wind Speed | 662.78 | 10.956 | 0.001 | 0.0929 |
| Avg Max Wind Speed | 1163.1 | 20.835 | 0.001 | 0.1630 |
